# Indirect cost with dementia: A Brazilian study

**DOI:** 10.1590/S1980-57642015DN91000007

**Published:** 2015

**Authors:** Ceres Eloah Lucena Ferretti, Ricardo Nitrini, Sonia Maria Dozzi Brucki

**Affiliations:** 1 RN, PhD Behavioral and Cognitive Neurology Unit, Department of Neurology, Hospital das Clínicas, University of São Paulo, SP, Brazil.; 2 MD, PhD Behavioral and Cognitive Neurology Unit, Department of Neurology, Hospital das Clínicas, University of São Paulo, SP, Brazil.; 3 MD, PhD Behavioral and Cognitive Neurology Unit, Department of Neurology, Hospital das Clínicas, University of São Paulo, SP, Brazil.

**Keywords:** dementia, economics, costs of illness

## Abstract

Costs with dementia have been the focus of research around the world and indirect costs to the caregiver appear in the literature as responsible for the greatest impact. In Latin American (LA) studies, indirect costs with dementia range from 60% to 75% of family income.

**Objective:**

To present preliminary results of the study "Description of the methods and cost analysis with dementia" currently being conducted at the Behavioral and Cognitive Neurology Unit of Hospital de Clínicas of University of São Paulo - HC-FMUSP.

**Methods:**

A cross-sectional study which, to date, includes interviews of 93 primary caregivers. The research protocol includes a sociodemographic questionnaire, the Functional Assessment Staging (FAST) scale, the Burden Interview (Zarit), an economic classification scale, and the Resource Utilization in Dementia (RUD) scale.

**Results:**

Monthly indirect costs were US$ 1,122.40, US$ 1,508.90 and US$ 1,644.70 stratified into mild, moderate and severe dementia, respectively. The projected annual indirect costs were US$ 13,468.80, US$ 18,106.80 and US$ 19,736.40, representing 69 to 169% of family income.

**Conclusion:**

This small sample showed that the impact of indirect costs with dementia in Brazil may be higher than that reported in other Latin American (LA) studies. These initial results may represent an important contribution for further research on costs with dementia in LA.

## Abstract (pt)

Custos com demência tem sido objeto de pesquisa em todo o mundo e os custos indiretos dos cuidadores aparecem na literatura como responsáveis por maior impacto.

**Objetivo:**

Apresentar resultados preliminares do estudo "Descrição dos métodos e análise dos custos com demência, que está sendo conduzido no ambulatório de Neurologia Cognitiva e do Comportamento do Hospital das Clínicas da Faculdade de Medicina da Universidade de São Paulo - HC-FMUSP.

**Métodos:**

Estudo de corte transversal que até o momento entrevistou 93 cuidadores primários. O protocolo de pesquisa inclui um questionário sociodemográfico, a escala de avaliação funcional (FAST), a escala de sobrecarga dos cuidadores (Zarit), uma escala de classificação econômica e a escala de utilização de recursos na demência (RUD).

**Resultados:**

Custos indiretos mensais variaram de US$ 1.122,40 a US$ 1.508,90 e US$ 1.644,70 estratificados pela gravidade da demência em leve, moderada e grave. Projeções anuais dos custos indiretos variaram de US$ 13.468,80 a US$ 18.106,80 e US$ 19.736,40 o que representou de 69% a 169% da renda familiar.

**Conclusão:**

Nossa pequena amostra mostrou que o impacto dos custos indiretos com demência no Brasil pode ser maior do que o encontrado em outros estudos latino americanos prévios. Esperamos que nossos resultados iniciais possam ser importantes para futures pesquisas sobre custos com demência na América Latina.

## INTRODUCTION

Costs with dementia have been the focus of research worldwide and informal care costs to caregivers appears to be responsible for the greatest impact.^1^

Dementia is considered an age-related disease and thus its prevalence and incidence of cases is higher among the elderly, i.e. persons aged 60-65 years or older. Currently, there is great concern over the global aging population, and the possibility of emergence of this type of neurodegenerative brain disorder.^2^ The United Nations - UN - estimates 700 million elderly people in the world today, a figure set to rise to 2 billion by 2050.3 In LA, Brazil's elderly represents 11% of its total population, and this can according to epidemiological data, have a profound impact on Latin American (LA) countries.^4,5^

The phenomenon of aging observed in recent decades, especially in developing countries, is undoubtedly a positive achievement, but leads to the need to prepare societies to adopt measures for controlling chronic non-communicable diseases - NCDs, including dementia.^2,3,6^ The economic impact of these diseases calls for organization of health systems in these countries, toward promoting quality during these additional years of life and adapting to the consequent change in the demographic and epidemiological profiles observed in recent decades.^2,6,7^

Dementia can be considered a multifactorial syndrome that rapidly or slowly imposes on individuals affected a serious loss of higher cortical functions.^8^ For disease course, affected individuals can expect 8-10 years or more of survival after diagnosis where this variation may be often associated with quality of care received by the patient. However, gradually, the person becomes totally dependent on others for the performance of their civil and everyday activities. Thus, at some point in the disease course, the need arises for specific pharmacological and non-pharmacological treatment given by a multidisciplinary team.^5,9,10^ In addition, informal caregivers (family member or close friend) or formal caregivers (paid caregiver or nursing home placement) may be required.^11,12^

Meeting these requirements renders the costs of the illness an important economic and social impact on monthly income of families, and exert a much larger social burden than some societies can afford, especially low income ones. With regard to expenditure on dementia, recent data from international organizations has revealed overall annual costs of US$ 604 billion, only 16% of which represents direct medical costs of health and social systems.^1^ Informal care costs represent the greatest burden, which in 2013 accounted for about US$ 216 billion, corresponding to 17.5 billion hours expended by informal caregivers on patients.^1,2^

In Europe, a recent review estimated a total annual cost of € 692 billion for the 19 subtypes of neurological disorders, including dementia. Of this total, € 105.2 billion was allocated to direct costs of their treatment.^13^ Regardless of the different economic and social models of countries of low, medium or high-income, the costs associated with dementia vary. These disparities have been attributed to the methodology used, such as the bases for calculating hours dispensed by the caregiver and lack of standardization of criteria on conceptual understanding and definitions of direct and indirect costs.^11^

According to Alzheimer Disease International - ADI, costs of dementia can be sub-classified as direct medical costs, direct social care costs and indirect costs.^14^ Direct medical costs refer to the medical care system, such as costs of hospital care, drugs and visit to clinics. Direct social care costs refer to formal services provided outside the medical care system and also out-of-pocket expenses of caregivers with items not covered by the local health system. There is some consensus among researchers concerning the difficulty making a clear distinction between social and medical care. Thus, direct costs are those related to social and public health coverages that are promoted by the Health System. Indirect costs include informal costs such as hours dedicated by the caregiver in meeting the needs of patients for basic activities of daily living (ADL), instrumental activities of daily living (IADL), supervision and total loss or reduction of formal working hours of caregiver productivity. Finally, indirect costs can be considered as the sum of informal costs + direct social costs to the caregiver.^14^ In this study, all indirect costs will be considered, excluding direct medical costs of the health system. As a basis for calculating indirect costs, some researchers adopt the minimum monthly wage in the country and an 8-hour working day to obtain the financial value of the hours spent on care.^1,15-17^

In LA, only a few studies have addressed costs with dementia^11,15,18^ and therefore the main objectives of the present study were to ascertain:

[1] the monthly/annual average indirect cost with dementia;
[2] the impact of this expenditure on the caregiver;
[3] whether severity of dementia translates to higher indirect costs; and
[4] Whether the financial burden correlates with higher overall burden levels for caregivers.

## METHODS

This is a cross-sectional observational study. The project "Description of Methods and Cost Analysis with Dementia" was submitted to the Research Ethics Committee of Hospital das Clinicas of the University of São Paulo - HC-FMUSP and approved under number 368.096/2013. In this paper, the preliminary results of the indirect costs of patients and their caregivers evaluated until 12/31/2013 are reported.

*Participants.* A convenience sample of outpatients followed at the neurology and cognitive behavior unit was assessed. All patients were previously assessed by a neurologist. At the end of the study, the sample will comprise 300 caregivers.

*Inclusion criteria.* Patients with a diagnosis of probable dementia at mild, moderate or severe stages;^19^ - Informal Caregivers spending time with the patient at least three times a week.

*Exclusion criteria.* Patients diagnosed with Mild Cognitive Impairment (MCI) and their caregivers.

*Professionals involved.* A nurse specialized in dementia and a neurologist.

*Measuring instruments.* A research protocol including:

- A semi-structured questionnaire, to collect sociodemographic and clinical status of patients and caregivers containing: Gender, age, education, retirement, income, economic status, kinship, marital status, place of residence of the patient, medical diagnosis, disease progression, co-morbidities, caregiver disease, use of medication prescribed by a physician. Direct monthly social care costs, out-of-pocket spending by caregiver on: drugs, medical appointments/health insurance, transportation, food, diapers, clothing, paid caregiver and other expenses. Hours spent by the caregiver on ADL, IADL, care and supervision.
- Functional Assessment Staging - FAST Scale, to stratify severity of dementia.^20^
- Economic Criterion Brazil scale, to identify the economic status of the caregiver.^21^
- Zarit Burden Interview scale, to determine the levels of caregiver burden.^22^
- Resource Utilization Dementia-RUD scale, to evaluate resources used in direct and indirect costs.^23^

**Procedures.** Caregivers were invited while waiting for medical appointments - waiting room - by the nurse responsible for the interviews, or were referred by the medical team after consultation. After presenting the study proposal to caregivers in detail, caregivers were given an Informed Consent Form and requested to sign this, retaining a copy.

To determine indirect costs with items not covered by the health system and paid out-of-pocket by the caregiver, the hours spent on care were calculated, taking as a basis, the current minimum wage (MW), updated annually, with the value thus obtained divided by a period of 30 days. The figure obtained was then divided by 8, considering a working day of 8 hours. The result was then multiplied by the number of hours of care dispensed by the caregiver to the patient thereby giving the value of the hours/day. Subsequently, the product was multiplied again to obtain the total monthly and projected annual costs. The calculation used in this study was based on a MW of R$ 678.00 (Brazilian currency), valid until 12/31/2013. All resultant values obtained in Brazilian currency (R$) were subsequently converted into U.S. currency ($) at the exchange rate of R$ 2.36:^1^ USD on 12/31/2013.

Dementia severity was obtained by the FAST classification and all patients were stratified into one of three stages: score 1-4="mild"; 5a-5e="moderate" and 7a-7f="severe". The scores on the burden interview lay in the ranges 0-20="little or no burden"; 21-40="mild to moderate"; 41-60="moderate to severe" and 61-88="severe". In this study, the scores corresponding to 0-20="little or no burden", were grouped under the "mild to moderate" rating. The economic classification was obtained according to the sum of the scores of declared items in each household. Thus, rating scores were: 42-46="A1"; 35-41="A2"; 29-34="B1"; 23-28="B2"; 18-22 "C1"; 14-17="C2"; 8-13="D"; 0-17="E" . The ratings "D" and "E" were grouped together in this study. For the final classification, this sum was added to the score corresponding to the declared level of patient education, where scores ranged from 0="illiterate/basic 1 incomplete"; 1="basic 1 complete/basic 2 incomplete"; 2="basic 2 complete/incomplete high school"; 4="complete high school/incomplete college"; 8="complete college". Caregiver education was obtained based on declared years of schooling.

**Statistics.** To compare categorical variables between groups, the Chi-square (χ^2^) test was performed while continuous variables were analyzed by one-way ANOVA, followed by Bonferroni analysis. To investigate correlation between financial burden, dementia severity, and caregiver burden, Spearman's R correlation analyses was performed. The level of significance was set at 0.05. Data were analyzed using the SPSS for Windows 20.0 software package.

## RESULTS

To date, a total of 93 caregivers have been interviewed. Demographic data on the three groups are shown in Table 1 and reached statistical significance for the variables: education, retirement, patient economic status, relationship to the patient, marital status and residing with the patient. Likewise, the clinical status of the sample - for patients and caregivers - showed significant results on: medical diagnosis, disease progression and comorbidities. There were no statistically significant differences in variables related to caregivers, as shown in Table 2. A greater percentage reduction/loss of productivity was observed among caregivers of patients classified as mild phase, but the total average reduction or loss of working hours was noted in the moderate stage of disease. Average monthly income ranged from 0 to 5 MW with the highest proportion (20.4%) of caregivers declaring a monthly income of 1 to 3 MW, and gross monthly amount of US$ 766.2 (SD ± 933.9) in the mild phase, US$ 825.9 (SD ± 558) moderate phase and US$ 684.5 (SD ± 450.3) in the severe phase, as shown in Table 3. The time spent by caregivers with variables ADL/IADL and supervision showed statistically significant results for the total number of hours spent per day, week and month. Total days spent per month, total cost per month and total cost per year (projection) were also statistically significant. Direct social care costs to the caregiver showed statistically significant results in all selected variables except "other expenses". The total indirect costs/month were $ 1,122.4, $ 1,508.9 and $ 1,644.7, and annual projections were $ 13,468.8, $18,106.8, and $ 19,736.4 US dollars in mild, moderate and severe stages, respectively (Table 4). There was some correlation between financial burden and caregiver burden in the severe stage and in total hours/month with ADL/IADL at the mild stage. Although a statistically significant difference was observed in almost all variables related to caregivers' out-of-pocket expenses, with the exception of "other expenses", these findings were not considered responsible for burden among caregivers. There was a weak positive correlation only for monthly medicine costs, consultations and medical health insurance. Significant, moderate, negative correlations were also found between the hours of care and caregiver burden (Table 5).

**Table 1:** Sociodemographic characteristics of the sample (n=93) stratified by severity of dementia (FAST).

Patient		Mild	Moderate	Severe	p-value
Gender M : F		19 (20%) : 21 (23%)	17 (18%) : 21 (23%)	7 (7.5%) : 8 (9%)	NS
Age	Mean (±SD) M : F	72 (±13) : 73 (±10)	70 (±12) : 72 (±18)	73 (±9) : 73 (±12)	NS
Education*	0	8 (9%)	11 (12%)	1 (1%)	
	1	12 (13%)	8 (9%)	5 (5%)	0.027**,^a^
	2	6 (6%)	3 (3%)	0 (0%)	
	4	9 (10%)	14 (15%)	3 (3%)	
	8	5 (5%)	2 (2%)	6 (6%)	
Retired		33 (36%)	32 (34%)	10 (11%)	<0.001^b^
Income	(0,1,2,3,4)^¶^ MW				NS
	0	16 (17%)	10 (11%)	4 (4%)	
	1	13 (14%)	17 (18%)	4 (4%)	
	2	2 (2%)	4 (4%)	2 (2%)	
	3	2 (2%)	2 (2%)	0 (0%)	
	4	7 (8%)	5 (5%)	5 (5%)	
Caregiver	Gender M : F	5 (5%) : 35 (38%)	5 (5%) : 33 (35%)	2 (2%) : 13 (14%)	NS
Age	Mean (±SD)	56 (±13)	53 (±13)	55 (±16)	NS
Education (year)	Mean (±SD)	8 (±6)	9 (±6)	9 (±5)	NS^a^
Economic classification					<0.001^b^
	A1	0 (0%)	0 (0%)	0 (0%)	
	A2	0 (0%)	2 (2.2%)	1 (1.1%)	
	B1	4 (4.3%)	2 (2.2%)	4 (4.3%)	
	B2	9 (9.7%)	10 (10.8%)	2 (2.2%)	
	C1	12 (12.9%)	19 (20.4%)	7 (7.5%)	
	C2	10 (10.8%)	3 (3.2%)	1 (1.1%)	
	D and E	5 (5.4%)	2 (2.2%)	0 (0%)	
Relationship					<0.001^b^
	Husband	3 (3.2%)	2 (2.2%)	1 (1.1%)	
	Wife	13 (14%)	12 (12.9%)	4 (4.3%)	
	Son or daughter	21 (22.6%)	20 (21.5%)	8 (8.6%)	
	Friend	3 (3.2%)	4 (4.3%)	2 (2.2%)	
	Other	0 (0%)	0 (0%)	0 (0%)	
Marital status					<0.001^b^
	Married	25 (26.9%)	29 (31.2%)	10 (10.8%)	
	Never married	9 (9.7%)	2 (2.2%)	3 (3.2%)	
	Divorced	2 (2.2%)	4 (4.3%)	0 (0%)	
	Separated	1 (1.1%)	3 (3.2%)	1 (1.1%)	
	Widowed	3 (3.2%)	0 (0%)	1 (1.1%)	
Number of children at home	Mean (±SD)	1 (±0.83)	1 (±0.84)	1 (0.80)	NS
Caregiver lives with patient		29 (31.2%)	28 (30.1%)	11 (11.8%)	<0.001^b^

M : F=Male : Female; NS=not significant;

* Education (0,1,2,3,4) where: 0=illiterate/basic I incomplete; 1=basic I complete/basic 2 incomplete; 2=basic 2 complete/incomplete high school; 3=complete high school/incomplete college; 4=complete college;

** Comparison between severity levels showed statistical significance (1≠3 and 2≠3);

a One way ANOVA for continuous variables, followed by Bonferroni test;

b ^χ2^ performed for nominal and categorical variables;

¶ Income (0,1,2,3,4) where: 0=up to 1 MW; 1=1 to 3 MW; 2=3 to 5 MW; 3=5 MW; 4=Not declared; MW=minimum wage. Note: statistical significance adopted was 0.05.

**Table 2:** Clinical Status of the sample (n=93) stratified by severity of dementia.

Patient		Mild (n e %)	Moderate (n e %)	Severe (n e %)	p-value
Diagnosis	AD	28 (30.1%)	31 (33.3%)	8 (8.6%)	<0.001*
	VD	3 (3.2%)	0 (0%)	0 (0%)	
	FTD	3 (3.2%)	2 (2.2%)	4 (4.3%)	
	LBD	0 (0%)	1 (1.1%)	0 (0%)	
	OD	6 (6.5%)	4 (4.3%)	3 (3.2%)	
Evolution (months)	Mean (±SD)	53 (±40)	58 (±41)	99 (±39)	<0.001**,^a^
Comorbidities	DM	12 (12.9%)	9 (9.7%)	2 (2.2%)	NS
	Hypertension	24 (25.8%)	25 (26.9%)	3 (3.2%)	0.011**,^a^
	VbD	7 (7.5%)	6 (6.4%)	4 (4.3%)	NS
	CvD	12 (12.9%)	18 (19.3%)	4 (4.3%)	NS
	Other	21 (22.6%)	13 (14%)	5 (5.4%)	NS
	Total by stage Mean (±SD)	1.8 (±1.2)	1.8 (±1.2)	1.1 (±1.3)	NS
Caregiver	Disease after taking on carer role	14 (15%)	15 (16%)	9 (10%)	
	How long after (in months) Mean (±SD)	29 (±24)	30 (±24)	35 (±15)	
	Use of medication prescribed by doctor	33 (36%)	29 (31%)	13 (14%)	

AD: Alzheimer’s disease; VD: Vascular dementia; FTD: Frontotemporal dementia; LBD: Lewy Body dementia; OD: Other dementia;

* χ^2^;

** Comparison between severity levels showed statistical significance (1≠3 and 2≠ 3);

a ANOVA; NS: not significant; VbD: Vascular brain disease; CvD: Cardiovascular disease.

**Table 3:** Reduction or loss of productivity of the sample of caregivers (n=93) stratified by severity of dementia

		Mild (n e %)	Moderate (n e %)	Severe (n e %)
Have a paid job		16 (17%)	14 (15%)	4 (4%)
Stopped/reduced workload		17 (18%)	10 (11%)	6 (6%)
If Yes, what reason	Reached retiring age	5 (5%)	1 (1%)	0 (0%)
	Early retirement	0 (0%)	0 (0%)	0 (0%)
	Was fired	0 (0%)	0 (0%)	1 (1.1%)
	Own problems with health	5 (5%)	0 (0%)	1 (1%)
	To care for the patient	2 (2%)	4 (4%)	1 (1%)
	Other	5 (5%)	5 (5%)	3 (3%)
	NA	21 (23%)	24 (26%)	8 (9%)
Working hours/week	Mean (±SD)	42 (±10)	41 (±13)	31 (±13)
Mean reduction or loss of total working hours (month)	Total hours	292,5	342	89
	Mean (±SD)	5 (±0.7)	10 (±0.7)	4 (±2)
	US$	350.7	410	106.7
	Retired caregiver	33 (36%)	29 (31%)	13 (14%)
Monthly income in MW (R$) Mean (±SD)	Up to 1 MW	2 (2.2%)	1 (1.1%)	0 (0%)
	1 to 3 MW	19 (20.4%)	14 (15.1%)	7 (7.5%)
	3 to 5 MW	14 (15.1%)	17 (18.3%)	6 (6.4%)
	>5 MW	5 (5.4%)	6 (6.4%)	2 (2.2%)
Monthly Income in US$	Mean (±SD)	766.2 (±933.9)	825.9 (±558)	684.5 (±450.3)

NA: not applicable; US$: American dollars; MW: minimum wage; R$: Brazilian currency.

**Table 4:** Indirect costs of caregivers sample (n=93) according to severity of dementia.

		Mild	Moderate	Severe	ANOVA p-value
Total hours/day with ADL/IADL and supervision – Mean (±SD)		10.6 (±8.5)	15.3 (±7.4)	17.9 (±7.5)	0.004*
Total hours/week with ADL/IADL and supervision – Mean (±SD)		73 (±60.3)	106 (±50.6)	125.5 (±52.5)	0.003*
Total hours/month with ADL/IADL and supervision – Mean (±SD)		323.3 (±260.6)	453.8 (±216.9)	538 (±224.9)	0.006*
Total days/month with ADL/IADL and supervision – Mean (±SD)		24.7 (±10.4)	29.3 (±3.7)	28.5 (±5.7)	0.028**
Total costs per month (US$)		309.5 (±291.9)	505.2 (±321.7)	567.6 (±322.5)	0.005*
Total projected costs per year (US$)		3714.0 (±3,503)	6062.4 (±3,860)	6811.2 (±3,873)	0.016*
Direct social care costs/month in US$ Mean (±SD)	Medication	67.5 (±70.6)	79.8 (±81.7)	125 (±145.5)	0.115
	Doctor visits/health insurance	59.3 (±107.9)	85.9 (±131)	108.4 (±126)	0.357
	Transportation	18.4 (±28.5)	46.4 (±55.1)	59.3 (±110)	0.034
	Food	287.2 (±167.7)	288.3 (±138)	305 (±146)	0.921
	Diapers	0.00 (0.00)	4.7 (±22)	110.2 (±95.5)	<0.001**
	Dressing	19.2 (±38.1)	28.7 (±43)	25.3 (±26.3)	0.549
	Other	15 (±27.7)	17.8 (±37.6)	23.6 (±37.3)	0.714
	Formal caregiver	8.5 (±38.6)	57.7 (±131)	215.2 (±346.6)	0.171
Total Direct social care + informal care + loss or reduction of productivity/month (US$)		1122.4	1508.9	1644.7	
Total projected indirect costs/year (US$)		13468.8	18106.8	19736.4	

ADL: Activities of daily living; IADL: Instrumental Activities of Daily Living;

* Comparisons were performed by the post hoc Bonferroni test and showed statistical significance at levels 1≠2 (mild ≠ moderate), 1≠3 (mild ≠ severe) and 2≠3 (moderate ≠ severe) of the FAST scale; US$: American dollars.

**Table 5:** Caregiver burden (ZBI) and correlation analyses (Spearman r) between indirect costs stratified by dementia severity (FAST).

		Dementia severity (FAST)					
		Mild (N=40)		Moderate (N=38)		Severe (N=15)	
Mean ZBI score of total caregivers sample (n=93) – Mean (±SD)		36 (±14)					
Number of caregivers by ZBI score (N:%)	Mild-moderate (21-40)	25: 62.5		**20: 52.6**		**10: 66.6**	
	Moderate-severe (41-60)	**14: 35.0**		15: 39.5		**4: 26.7**	
	Severe (61-88)	**1: 2.5**		**3: 7.9**		1: 6.7	
		**40:100.0**		**38:100.0**		**15:100.0**	
		**Rho**	**p value**	**Rho**	**p value**	**Rho**	**p value**
Total hours/Day with ADL/IADL and supervision		0.287	0.072	0.082	0.626	–0.409	0.130
Total hours/week with ADL/IADL and supervision		0.296	0.064	0.109	0.517	–0.409	0.130
Total hours/month with ADL/IADL and supervision		0.366	0.020*	0.111	0.508	–0.409	0.130
Total days/month with ADL/IADL and supervision		0.142	0.381	0.219	0.187	–0.336	0.221
Total costs per month		0.300	0.060	0.158	0.344	–0.603	0.017*
Total costs projected per year		0.300	0.060	0.160	0.337	–0.603	0.017*
Direct social care costs (month)	Medication	–0.095	0.559	0.036	0.039*	–0.138	0.623
	Doctor visits/health insurance	–0.039	0.812	0.373	0.021*	–0.138	0.623
	Transportation	–0.175	0.280	–0.078	0.643	0.339	0.216
	Food	0.029	0.860	0.123	0.461	0.057	0.839
	Diapers	–	–	0.006	0.973	–0.069	0.806
	Dressing	–0.074	0.651	–0.222	0.180	0.429	0.110
	Other	0.020	0.902	–0.073	0.661	–0.032	0.911
	Formal caregiver	–	–	–	–	–	–
Total direct social care costs + informal care costs + loss or reduction of productivity/month (US$)		**1122.4**		**1508.9**		**1644.7**	
Total indirect projected costs/year (US$)		**13468.8**		**18106.8**		**19736.4**	

ADL: Activities of Daily Living; IADL: Instrumental Activities of Daily Living;

* significant correlation; ZBI: Zarit Burden Interview; N:%: number of caregivers:percentage.

## DISCUSSION

The preliminary results of this study, as expected, showed a significant impact of indirect costs with dementia in Brazil. Findings from recent studies have associated overheads of indirect costs with the advancement of the disease and the present study found the same result.^15,17^

Some sociodemographic variables of patients and caregivers were similar to those found in other studies, maybe owing to cultural identities, for example: gender, age, marital status, education, relationship, retirement, economic status and monthly income of patients.^5,9,10,11^

Regarding the clinical status of patients and caregivers, some significant disparities were found, including higher prevalence of AD, duration of the disease and the presence of comorbidities in patients, the most prevalent being hypertension, pointing to the importance of primary care actions. Among caregivers, it was noted that their clinical status can change an average of 2-3 years after taking on the role of carer. There was a high prevalence of emotional stress on caregivers, as observed in other studies. Few caregivers were classified as having low levels of stress as compared to other caregivers of patients without dementia. A LA study found a higher frequency of depression in caregivers than non-caregivers of 44% and 27%, respectively,^24^ with anxiety (96%) and depression (100%) + family dysfunction (26.5%) in 47% of primary caregivers of patients with dementia.^25^ We found a 38% rate of reports of various diseases in caregivers that started after taking responsibility for the care of their ill relative, with a higher percentage of reports (16%) observed at the moderate stage. The loss or reduction of hours of productivity was found not to increase with severity of the disease and it was noted that greater reduction or loss of formal working hours occurred at mild and moderate stages. This finding may be due to the small sample of patients in the severe phase, but also suggests that the search for medical diagnosis and treatment in the early stages may explain the higher consumption of caregivers hours as well as the reduction or even loss of productivity.^26^ In the moderate stage, the need for a caregiver generates higher consumption of hours of informal care and expenses related with direct social care costs and our findings corroborate other studies showing increased burden as the disease progresses.^11,15,18,27^ The reduction or loss of hours of productivity observed at the severe stage can be viewed as the consequence of the high number of hours spent per month associated with the severity of dementia.^15^

Perhaps, indirect costs to the caregivers can be minimized by the establishment of pharmacological and non-pharmacological therapies that may promote greater flexibility and allow time for the caregivers to perform other tasks.^28^ In this regard, awareness and education initiatives can be very useful in low and middle income countries.^5^ Nevertheless, it should be noted that the average number of working hours reduced or lost per month due to care is high, and the values reached by the calculations can exceed the monthly income of many families, as was shown in this study. A high percentage of caregivers reported income of 1 to 3 MW, and declared income, in some cases, fell below of the amounts estimated for indirect costs. Our estimates showed that these costs exceeded, in some cases, the declared family income at rates of 69% to 106% in the mild stage; 89% to 136% in the moderate stage and 106% to 169% in the severe stage. Recent LA studies have found committed family incomes of 60%,^18^ 61%,^15^ and 66%-75%.^11^ The direct social care costs of the caregivers in the present study were greater than the monthly income of some caregivers in some cases, totaling US$ 462.15 at mild, US$ 593.70 at moderate, and US$ 970.40 at severe stages. The higher spending of caregivers seen in the severe stage of the disease is in agreement with the finding of other authors, being attributed to the greater complications observed at later stages.^11, 15,26^

The average hours of care, weekly and monthly, was higher than hours seen in other studies and contributed to a greater caregiver burden than financial burden, suggesting that actions of support and education, as proposed in other studies, should be sought to reduce the burden of caregivers.^5,9,10^ However, comparison of these results were hampered by methodological differences in LA, lack of stratification of severity of dementia observed and by the fact that only indirect costs were addressed in the present study. Also, an international comparative cost evaluation was not conducted. However, some studies in LA found lower hours of care than that observed in this study.^2,11,15,18^ In Europe, findings from a study that estimated hours of care stratified by disease severity were similar to this study results, especially at the mild stage of dementia.^26^ We found average proportion of 29%, 63% and 75% of weekly hours; 45%, 63% and 75% of monthly hours; and a total average days per months corresponding to 82.3%, 98% and 95%, in mild, moderate and severe phases, respectively.

The high number of hours reported here could be related to exceeding of caregiver limits and not to increased demands for end-of-life care, since a high percentage of patients were at mild and moderate phases.^27^

These findings reveal the extent to which these caregivers fail to earn an income owing to premature withdrawal from the labor market, and of the risk of themselves becoming dependent on the health system given that diseases such as diabetes mellitus, depression and hypertension are also diseases with high morbidity, responsible for other NCDs. The lack of social structure has led to greater isolation of individuals that, with no alternatives, are forced to leave their work and life plans, to take care of their sick relative. In some cases, those with no monthly income become dependent on the pension or allowance of the patient or rely on the help of others to ensure survival of the family.^29^ In Brazil, initiatives include supportive measures and education of professionals and caregivers with some positive results in reducing the burden of care.^5,28,30^ Further research and multicenter studies to compare the results obtained here are necessary. This small sample showed that the impact of indirect costs with dementia in Brazil may be higher than other previous LA studies. These initial results may make an important contribution to future research about costs with dementia in LA.

The main limitation of this study was its cross-sectional design. Most studies assessing the direct and indirect costs of dementia in high income countries have involved longitudinal studies. Recent literature on this issue alerts the attention of researchers to the need for systematic actions that focus on early diagnosis and treatment. This systematization of actions has been considered a possible approach for reducing the direct and indirect costs associated with the disease.

In our milieu, there remains much to investigate and achieve in the sphere of non-communicable diseases Despite the limitations noted , this is the first Brazilian study to perform the monetary calculation of indirect costs associated with dementia, where the next step is to continue the research presented employing an appropriate methodological design.
